# Immune Response Gene Expression in Colorectal Cancer Carries Distinct Prognostic Implications According to Tissue, Stage and Site: A Prospective Retrospective Translational Study in the Context of a Hellenic Cooperative Oncology Group Randomised Trial

**DOI:** 10.1371/journal.pone.0124612

**Published:** 2015-05-13

**Authors:** George Pentheroudakis, Georgia Raptou, Vassiliki Kotoula, Ralph M. Wirtz, Eleni Vrettou, Vasilios Karavasilis, Georgia Gourgioti, Chryssa Gakou, Konstantinos N. Syrigos, Evangelos Bournakis, Grigorios Rallis, Ioannis Varthalitis, Eleni Galani, Georgios Lazaridis, George Papaxoinis, Dimitrios Pectasides, Gerasimos Aravantinos, Thomas Makatsoris, Konstantine T. Kalogeras, George Fountzilas

**Affiliations:** 1 Department of Medical Oncology, Ioannina University Hospital, Ioannina, Greece; 2 Department of Pathology, Aristotle University of Thessaloniki School of Medicine, Thessaloniki, Greece; 3 Laboratory of Molecular Oncology, Hellenic Foundation for Cancer Research, Aristotle University of Thessaloniki School of Medicine, Thessaloniki, Greece; 4 STRATIFYER Molecular Pathology GmbH, Cologne, Germany; 5 Department of Medical Oncology, “Papageorgiou” Hospital, Aristotle University of Thessaloniki School of Medicine, Thessaloniki, Greece; 6 Section of Biostatistics, Hellenic Cooperative Oncology Group, Data Office, Athens, Greece; 7 Oncology Unit GPP, "Sotiria" General Hospital, Athens School of Medicine, Athens, Greece; 8 Department of Clinical Therapeutics, “Alexandra” Hospital, University of Athens School of Medicine, Athens, Greece; 9 Oncology Department, General Hospital of Chania, Crete, Greece; 10 Second Department of Medical Oncology, “Metropolitan” Hospital, Piraeus, Greece; 11 Oncology Section, Second Department of Internal Medicine, “Hippokration” Hospital, Athens, Greece; 12 Second Department of Medical Oncology, “Agii Anargiri” Cancer Hospital, Athens, Greece; 13 Division of Oncology, Department of Medicine, University Hospital, University of Patras Medical School, Patras, Greece; 14 Translational Research Section, Hellenic Cooperative Oncology Group, Data Office, Athens, Greece; The First Affiliated Hospital of Nanjing Medical University, CHINA

## Abstract

**Background:**

Although host immune response is an emerging prognostic factor for colorectal cancer, there is no consensus on the optimal methodology, surrogate markers or tissue for study.

**Patients and Methods:**

Tumour blocks were prospectively collected from 344 patients with stage II/III colorectal cancer (CRC) treated with adjuvant chemotherapy. Whole section lymphocytic infiltration was studied along with mRNA expression of CD3Z, CD8, CD4, CXCL9, CXCL13, IGHM, FOXP3, SNAI2 and ESR1 by qRT-qPCR in tissue microarray (TMA) cores from the centre of tumour, invasive margin and adjacent normal mucosa.

**Results:**

Lymphocytic infiltration, deficient MMR (10.9%), KRAS (40.7%) and BRAF (4.9%) mutations or single mRNA gene expression were not prognostic. Tumour ESR1 gene expression (Hazard Ratio [HR] for relapse 2.33, 95% CI 1.35-4.02; HR for death 1.74, 95% CI 1.02-2.97) and absence of necrosis (HR for relapse 1.71, 95% CI 1.05-2.71; HR for death 1.98, 95% CI 1.14-3.43) were adverse prognostic features. We used CD3Z and CD8 expression in order to devise the mRNA-based Immune Score (mIS) and proceeded to partitioning analysis in 267 patients, with age, stage, tumour site (Right vs Left CRC), KRAS mutation and tumour mIS as input factors. Only in patients with stage III right-sided colon cancer, a low immune response was associated with inferior disease-free survival (mIS-low, HR for relapse 2.28, 95% CI 1.05-8.02). No prognostic significance was seen for tumour mIS in any other stage or site of CRC, or for a similar mIS score derived from adjacent normal mucosa. Independent adverse prognostic significance was retained in multivariable analysis for absence of necrosis, tumour ESR1 expression in all patients and low tumour mIS in stage III right-sided CRC.

**Conclusions:**

In localised CRC, mRNA-based CD3Z/CD8 profiling of tumour immune response may have stage, site and tissue-specific prognostic significance, along with ESR1 expression.

**Trial Registration:**

ANZCTR.org.au ACTRN12610000509066

## Introduction

Colorectal cancer (CRC) is the second cause of cancer death in the Western world. In Europe, 376000 new cases of colorectal cancer are diagnosed each year, with mortality observed in 204000 patients[1]. Despite advances in adjuvant systemic therapies, half of the patients who undergo resection experience malignant relapse. Over the last decade, published evidence suggests that outcomes in colorectal cancer are not solely dependent on tumour characteristics, but also on the interactions between host and malignancy. The host immune response against the tumour is a finely tuned, multicellular and humoral reaction, emerging as an important factor for shaping the tumour-microenvironment interactions and for defining patient prognosis[2]. Controversy still persists on the ideal immune response markers to be followed, the tissue for study, the optimal methodology to be used and most importantly, the independent prognostic significance of tumour-associated immune reaction in relation to other clinicopathological and molecular variables[3]. The factors modulating the tumour-associated immune response, the significance of the immune response in adjacent normal colonic mucosa and the site specificity of the immune reaction need to be further explored.

Several hypotheses have been generated on the impact of molecular and biological processes on the intensity and nature of the host immune response. Mismatch repair (MMR) deficiency results in accumulation of mutations and consequently of aberrant tumour proteins, eliciting an intense host immune reaction[4]. In contrast, the Epithelial-Mesenchymal Transition (EMT) may down-regulate malignant expression of epithelial neoantigens, thus blunting the immune reaction[5]. Estrogen receptor (ER) signalling via ESR1 and 2 also regulates the tumour-promoting and tumour-suppressive effects of the immune response in several human malignancies[6, 7]. Finally, there is still dubiety on whether the distinct embryological, perfusion, physiological, molecular and phenotypic characteristics of left versus right-sided CRC impact on the host immune reaction and its tumour-promoting versus tumour-suppressive effects[8].

In view of the above, we sought to retrospectively study clinicopathological characteristics, lymphocytic infiltration, gene mutations and gene expression profiles of immune cells, EMT markers, ER in prospectively collected tumour paraffin blocks of patients with resected stage II/III colorectal cancer enrolled in a randomised trial of adjuvant FOLFOX versus XELOX chemotherapy. Our objectives were to a) identify clusters of combined immune mRNA markers from tumour or adjacent normal colonic mucosa with prognostic significance b) study the correlation of the latter with a plethora of clinical, pathological and molecular variables and c) assess the impact of tumour stage and site on the prognostic utility of host immune response.

## Patients and Methods

From the 14^th^ of October 2005 until the 22^nd^ of January 2008 a total of 441 patients with high risk stage II (perforation or obstruction at diagnosis, T4, grade 3, lymphatic or vascular invasion, less than 13 lymph nodes removed) or stage III CRC were enrolled in the HE6C/05 prospective phase III trial randomising between 12 cycles of FOLFOX (folinic acid 200 mg/m2 intravenously, intravenous bolus at 400 mg/m2 and infusional 5-FU at 2400 mg/m2 over 46 hours, oxaliplatin at 85 mg/m2 over two hours intravenously every two weeks) versus 8 cycles of XELOX (capecitabine at 2000 mg/m2 po daily for 14 days, oxaliplatin at 130 mg/m2 intravenously on day 1 every three weeks) adjuvant chemotherapy[9]. After the completion of adjuvant chemotherapy, patient evaluation was done by means of physical examination, CEA levels every three months and CT of chest/abdomen/pelvis every four months.The clinical protocol was approved by Institutional Review Boards (IRBs) in Ioannina University Hospital, “Papageorgiou” Hospital, University Hospital of Patras, “Henry Dunant” Hospital, “Agii Anargiri” Cancer Hospital, “Alexandra” Hospital, “Sotiria” Hospital, “Attikon” Hospital and by the National Organization for Medicines (14/10/2005). Before randomization, each patient provided study specific written informed consent for participating in the trial and optionally a separate informed consent for providing biological material for research purposes. All clinical investigations related to the present study have been conducted according to the principles expressed in the Declaration of Helsinki. The HE6C05 trial was included in the Australian New Zealand Clinical Trials Registry and allocated the following Registration Number: ANZCTR 12610000509066. The trial was registered retrospectively as at the time of trial initiation there was no requirement for clinical trial international registration by the Greek legislation. We registered our trial after it became required by the International Committee of Medical Journal Editors (ICMJE). The authors confirm that all ongoing and related trials for this drug/intervention are registered.

Histological examination of whole sections was performed by an experienced pathologist (G.R). Sections were reviewed for the presence of tumour, surface ulceration, invasive depth and anatomic component, desmoplastic stroma, bulk necrosis, hemorrhagic infiltrates and extracellular mucus. H&E sections were marked for (a) tumor dense areas in the centre and in the invasive margin (IM) of the lesion avoiding to the extent possible the non-cellular elements described above; and (b) where present, normal mucosa with as much as possible representation of the epithelial compartment. From these areas tissue cores were procured for tissue microarray (TMA) construction. TMAs were constructed with the Alphelys Minicore 3 Tissue Microarray system (Plaisir, France), including three 1.0 mm tissue cores per tumour from the areas marked on the H&E sections and three cores from the adjacent morphologically normal mucosa, where available. Tumour and normal cores were loaded on the same TMA. In total, 32 such low-density TMAs were constructed including 344 tumors and 272 normal mucosa cases. TMA sections were reviewed for tumour cell content (TCC), which was assessed collectively for all 3 cores of each tumour.

Lymphocytic infiltrates were recorded separately for tumour and morphologically normal tissue compartments in whole sections. Lymphocytic infiltrates for each tumour were identified at 400X magnification and assessed at 100X semi-quantitatively; these were graded as absent (none), focal (up to 5% of tumour area), moderate (6–20%) and extensive (>20%). This classification was applied for tumours and normal compartment in whole sections.

### Immunohistochemistry (IHC) for Mismatch Repair (MMR) Proteins

IHC was carried out in cores from 2 μm thick TMA sections with the following antibodies and conditions: MLH1, clone ES05 (Monosan, Uden, Netherlands) at 1:80 dilution; MSH2, clone 25D12 (Novocastra/Leica Microsystems, Wetzlar, Germany) at 1:40 dilution; MSH6, clone EP49 (DAKO, Glostrup, Denmark) at 1:60 dilution; and, PMS2, clone M0R4G (Novocastra/Leica Microsystems) at 1:50 dilution. All tests were performed using a Bond Max autostainer (Leica Microsystems) with diaminobenzidine as chromogen for protein-antibody complex visualization. Stains were evaluated by two pathologists (G.R. and E.V.) for all tumour and normal cores, along with external controls for assessing method performance. Each core was evaluated for nuclear staining intensity and distribution of positive cells at 200X and 400X magnification. Cases were considered as pMMR (proficient MMR) if any degree of nuclear expression was observed for all four proteins in the neoplastic cells and dMMR if no IHC nuclear expression was seen for any of the four proteins.

### Tumor genotyping for KRAS/BRAF mutations

DNA extraction was performed from 8 μm TMA sections, for tumours with TCC >30% in all 3 cores (n = 330). Following deparaffinization, the VERSANT Sample 1.0 Reagent Kit (Siemens Healthcare Diagnostics, Tarrytown, NY) was used for manual magnetic DNA isolation, according to the manufacturer’s instructions. Tumour KRAS mutations (exons 2, 3, 4, coding exons 1, 2, 3) and BRAF V600E (exon 15) were assessed with dd-sequencing on nested PCR amplicons, as shown in S1 Table. Primers were located in adjacent introns spanning the entire coding regions of interest. Nested primers were coupled with universal M13 forward and reverse primers at the 5’-end. Sense and antisense sequencing was performed using M13 forward and reverse primers in 10 μl reactions with the Big Dye Terminator kit v.1.1 (Applied Biosystems/Life Technologies, Paisley, UK). Products were visualized upon capillary electrophoresis in an ABI3130XL genetic analyzer, base called and further analysed with the Sequencing Analysis version 5.2 software (Applied Biosystems). In total, 302 tumours were informative for KRAS and BRAF exon 15 mutation status.

### RNA isolation from FFPE tissue and quantitative reverse transcription-polymerase chain reaction (qRT-PCR) assessment

Total RNA was also extracted from TMA cores (approximately 15–18 cores for each sample, 2/3 of which were from tumour centre and 1/3 from the invasive margin) with the TCC criteria applied above, using a standardized isolation method for total RNA from FFPE tissue, based on silica-coated magnetic beads (VERSANT Tissue Preparation Reagents, Siemens Healthcare Diagnostics, Tarrytown, NY), as previously described in detail[10]. In the last step DNaseI digestion was performed.

qRT-PCR Primers and 5’-FAM–3’-TAMRA-labeled hydrolysis probes were selected using Primer Express Software, Version 2.2 and 3 (Applied Biosystems, Life Technologies, Karlsruhe, Germany) according to the manufacturer’s instructions and were controlled for single nucleotide polymorphisms. All primers, probes and amplicons were checked for their specificity against nucleotide databases at NCBI using Basic Local Alignment Search Tool (BLAST). Primers and probes were purchased from Eurogentec S.A. (Seraing, Belgium). For each gene primer/probe set the amplification efficiency was tested, aiming to reach comparable efficiency of >90% (efficiency range from 91 to 100%). In case the efficiency did not reach such levels, a new primer/probe set was designed and tested. Primers and hydrolysis probes were diluted to 100 μM, using a stock solution with nuclease-free water (Qiagen, Hilden, Germany). qRT-PCR was applied for the relative quantification (RQ) of the immune response genes CD3Z, CD8, CD4, CXCL9, CXCL13, IGHM and FOXP3, the EMT transcription factor SNAI2 and the estrogen receptor a gene (ESR1). For sequences see S2 Table, for functional roles of the studied genes in immune response, see S4 Table. For PCR, 0.5 μM of each primer and 0.25 μM of each probe were used. All quantitative reverse-transcription PCRs were performed in triplicates using the SuperScript III Platinum One-Step qRT-PCR kit with ROX (Invitrogen/Life Technologies, Darmstadt, Germany) according to the manufacturer’s instructions. Experiments were performed on a Stratagene Mx3005p (Agilent Technologies, Böblingen, Germany) with 30 min at 50°C and 2 min at 95°C followed by 40 cycles of 15s at 95°C and 30s at 60°C. When available, paired tumour and normal samples were included in the same run. Samples were considered eligible for further investigation when the Cq (cycle quantification) values of the housekeeping gene were <32 (triplicate mean values). Relative expression levels (relative quantification, RQ) of the target transcripts were calculated as (40 –[mean Cq target gene—mean Cq housekeeping gene]) to yield positively correlated numbers and to facilitate comparisons. RQ expression of the genes under study was obtained for tumour tissue in 286 cases and for matched adjacent normal mucosa in 144 cases. The RQ values from the tumour tissue reflected mRNA levels from both the tumour centre and the IM.

### Statistical Analysis

The primary endpoint of the HE6C05 trial was Disease-Free Survival. OS was measured from the date of randomization to the date of patient’s death or last contact, while DFS was measured from the date of randomization to documented first recurrence or death without prior documented recurrence. Surviving patients were censored at the date of last contact. Time to event distributions were estimated using Kaplan-Meier curves and compared using the log-rank test. For all univariable tests significance level was set at α = 0.05. Continuous variables were presented as median with the corresponding range and categorical variables as frequencies with the respective percentages. Chi-Square or Fisher’s exact test and the non-parametric Mann-Whitney test were used for comparing patient and tumour characteristics. Since this is a retrospective translational research project, not a priori defined in the clinical trial protocol, no formal statistical hypothesis was available.

We applied Receiver Operating Characteristic (ROC) curve analysis with the 3-year disease-free survival status as an endpoint in order to select cut-offs with optimal sensivitity and specificity for categorisation of all continuous study parameters to positive versus negative. We used the 3-year DFS benchmark because of its clinical value, the higher number of relapses compared to the number of deaths, (providing more events and increasing the statistical power) and its established correlation with 5-year OS[11].When the ROC analysis did not yield satisfactory cut-offs we examined the quartile distribution values of the parameter (25th, 50th and 75th percentile) for prognostic significance (data not shown).

Two-way clustering analysis was performed for combinations of the seven immune response genes (CD3Z, CD8, CD4, CXCL9, CXCL13, IGHM and FOXP3). Gene expression was examined and plotted by correlation/similarity of expression (columns) and by tumour cases (lines). The “two-way clustering” is the joint presentation on the same plot of the clusters derived for the patients and genes, after applying the same clustering method on patients and genes separately. A color map is automatically created with two dendrograms, a row (patients) and a column (genes). The resulting clusters were examined for a) variability of gene expression between patient groups, b) optimal separation of patient groups in terms of gene expression geometric distance scale and c) prognostic significance. The 2-cluster profiler based on the mRNA expression of tumour CD3Z and CD8 was defined as the mRNA-based Immunoscore (mIS).

A decision tree model, using recursive partitioning in JMP, was run for the 3-year disease-free survival status with the following input parameters: AJCC-UICC tumour stage, site (ascending colon, hepatic flexure, proximal 2/3 of transverse colon: Right vs Left colon/rectum), patient age (<65 or >65), KRAS mutation status and the mRNA-based Immunoscore (mIS). At each node, the optimal classification variable proposed by the software was chosen and applied. As the HE6CO5 trial did not show any survival difference between the FOLFOX and XELOX arms, we did not include the adjuvant therapy in the model.

We applied multivariable Cox regression analysis according to the Bald backwards procedure by using as input factors tumour ESR1 gene expression (positive vs negative), tumour KRAS gene mutation status (wild-type vs any mutation in exons 2, 3, 4), presence of necrosis at histology, histological grade, patient age (<65 vs >65), gender, performance status (ECOG 0 vs 1 or higher) and the complex partitioning parameter Stage_Site_tumour mIS. The latter was composed out of the following parameters: Stage II vs. III; right vs. left location; and, CD3/CD8 mRNA high (high expression for both in the cluster) vs. low (either low or both low in the cluster) expression. Cross comparisons for each category in the 3 parameters resulted in 8 categories in the combined variable, e.g., stage II, right location, CD3/CD8 low; stage II, right location, CD3/CD8 high; stage II, right location, CD3/CD8 low; stage II, left location, CD3/CD8 low; stage II, right location, CD3/CD8 low; stage II, left location, CD3/CD8 high; similarly for combinations with stage III. P-values for removal in the analysis were set at α = 0.15.

Multiple imputation with the MCMC algorithm and 100 imputed data sets targeting the mRNA parameters and having as auxiliary variables the basic patient demographics and tumour characteristics was performed in order to test the validity of the main findings regarding ESR1 and the complex parameter. The SAS software was used for all statistical analyses (SAS for Windows, version 9.3, SAS Institute Inc., Cary, NC) except for the decision tree model for which the JMP software was used (version 10.0, SAS Institute Inc.).

## Results

### Patient and Tumour Characteristics

**Fig 1:**
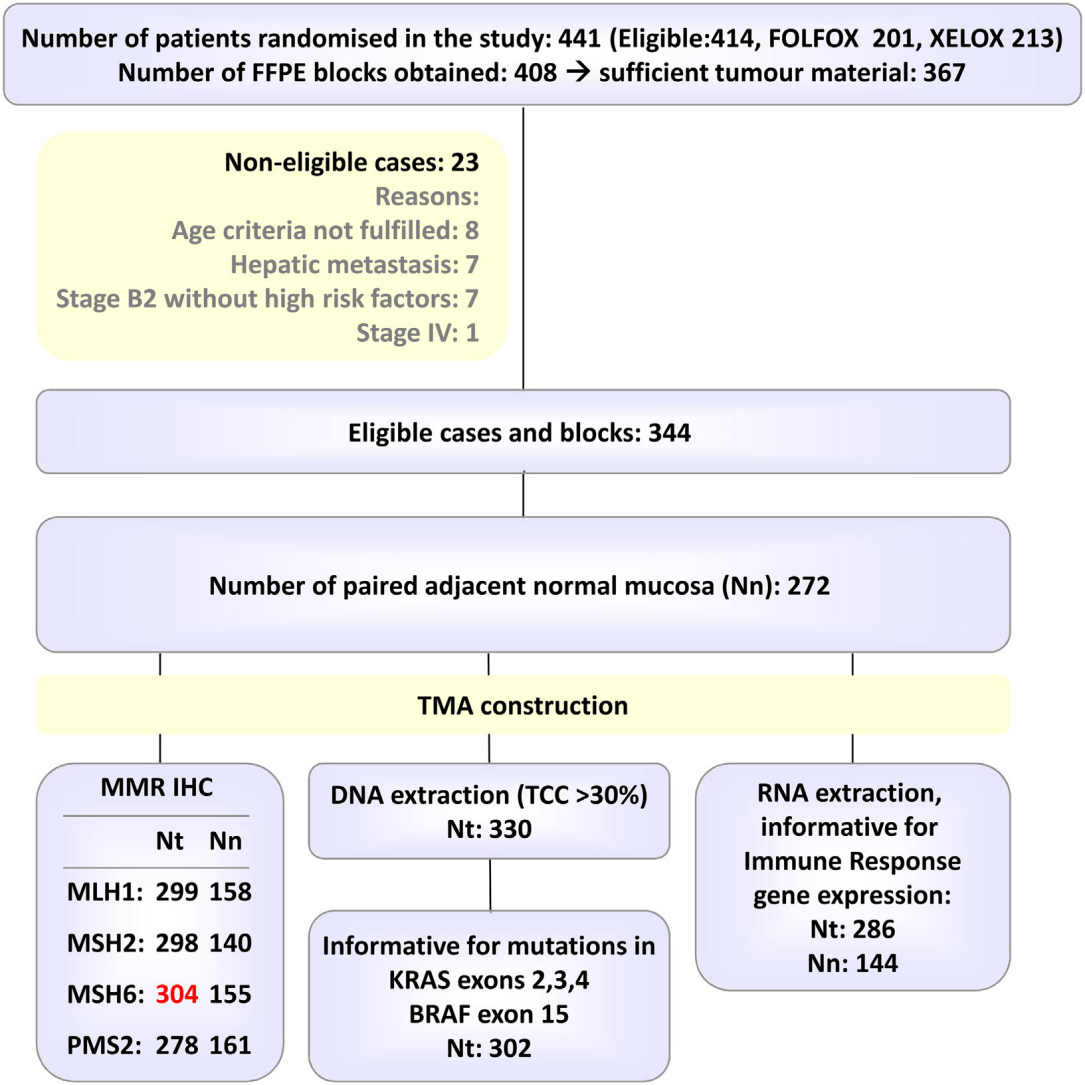
REMARK diagram.

Formalin-fixed paraffin-embedded tumour blocks were prospectively collected in 344 cases (Fig 1), which were used for all analyses.Patients hadresected high-risk stage II (33.9%) or stage III (66.1%) colon (73.1%) or rectal (26.9%) adenocarcinomas. A right-sided tumour (R CRC) had been diagnosed in 31.6% of patients. Poor tumour differentiation was observed in 19.5% of cases, while mucinous histology in 29.8%. Histologic evidence of necrosis was present in the majority of specimens (81.8%). Among the 304 tumours immunohistochemically studied for MMR proteins, MMR deficiency was observed in 33 tumours (10.9%). Among the 302 tumours examined for gene mutations, KRAS mutations were found in 40.7% and BRAF mutations in 4.9%. At a median follow-up of 74.7 months, 98 patients (28.5%) had relapsed and 73 (21.2%) had died. Basic patient and tumour demographics are summarised in Table 1. We observed no significant differences in patient and tumour characteristics between the clinical trial population (n = 441), the translational project population (n = 344) and the population with IR mRNA data (n = 286).

**Table 1:** Patient and Tumour Demographics in all cohorts.

	N = 441	N = 344	N = 286	2-sided P Value	2-sided P Value
	Study Population	Tumour TMA Population	Tumour mRNA Population	344 vs. excluded	286 vs. excluded
**Age (years)**					
Median (range)	63 (24–75)	63 (24–75)	64 (24–75)	0.832	0.880
<65	270 (61.2%)	207 (60.1%)	169 (59.1%)	0.394	0.212
≥65	171 (38.7%)	137 (39.9%)	117 (40.8%)		
**Gender**					
Male	244 (55.2%)	196 (57.0%)	156 (54.6%)	0.190	0.653
Female	197 (44.7%)	148 (43.0%)	130 (45.6%)		
**Performance Status**					
0	400 (90.6%)	314 (92.9%)	264 (92.4%)	0.888	0.844
1	31 (6.9%)	24 (7.1%)	21 (7.2%)		
NR	10	6	1		
**Histological Grade**					
1–2	355 (80.4%)	277 (80.5%)	229 (80.1%)	0.981	0.757
3	86 (19.5%)	67 (19.5%)	57 (19.8%)		
**Mucinous histology**	111 (25.2%)	90 (29.8%)	74 (25.8%)	0.268	0.665
**Adjuvant therapy**					
FOLFOX	218 (49.5%)	170 (49.4%)	143 (50.0%)	0.991	0.746
XELOX	223 (50.7%)	174 (50.6%)	143 (50.0%)		
**Primary Site**					
Cecum, Asceding, Hep Flexure, Transverse	136 (31.1%)	108 (31.6%)	89 (35.6%)	0.652	0.860
Splenic Flexure, Descending, Sigmoid	182 (41.6%)	142 (41.5%)	124 (40.9%)		
Rectum	120 (27.4%)	92 (26.9%)	71 (23.4%)		
NR	3	2	2		
**AJCC-UICC stage**					
II	147 (34.4%)	115 (33.9%)	97 (34.4%)	0.668	0.986
III	280 (65.6%)	224 (66.1%)	185 (65.6%)		
NR	14	5	4		
**KRAS mutations**					
Wild-type	186 (58.3%)	179 (59.3%)	167 (59.2%)	0.147	0.499
Exon 2 mutation	111 (34.8%)	103 (34.1%)	97 (34.4%)		
Exon 3 or 4 mutation	22 (6.9%)	20 (6.6%)	18 (6.4%)		
NR	122	42	4		
**BRAF V600E mutations**					
Wild-type	306 (69.3%)	287 (95.0%)	270 (95.1%)	0.320	0.546
Mutated	15 (3.3%)	15 (5.0%)	14 (4.9%)		
NR	120	42	2		
**Mismatch Repair IHC status**					
Proficient MMR	288 (65.4%)	271 (89.1%)	253 (88.8%)	0.964	0.535
Deficient MMR	35 (7.8%)	33 (10.9%)	32 (11.2%)		
NR	118	40	1		
**Necrosis at histology**					
Yes	269 (60.9%)	252 (81.8%)	231 (85.9%)	0.285	0.931
No	44 (9.9%)	56 (18.2%)	38 (14.1%)		
NR	128	36	17		
**Ulceration at histology**					
Yes	69 (15.6%)	67 (25.3%)	61 (25.5%)	0.249	0.369
No	212 (48%)	198 (74.7%)	178 (74.5%)		
NR	160	79	47		

NR, not reported; MMR, mismatch repair. *The above table contains the description of the population sets used or not used for the analysis. The last two columns contain the comparison p-value (chi-square of K-W, where appropriate) among cohorts of excluded and included patients*.

The mean percentage of tumour area with tumour-infiltrating lymphocytes (TIL) was 9.3%, in representative whole sections. Most cases had either weak (TIL 1–5% in 37.9%) or moderate (TIL 6–20% in 57.4%) lymphocytic infiltration, with very few cases showing an intense lymphocytic reaction [lymphocytic infiltration >20% of tumor area in 14 cases (4.7%)]. A similar lymphocytic reaction was present in both the centre of the tumour and at the periphery (IM), in almost all cases. However, in the absence of IHC phenotypic characterisation of TILs, we did not proceed to further analyses of TIL association with other variables or with prognosis by microenvironment localization (centre of the tumor vs IM lymphocytes). An intense fibrotic reaction, defined as >20% of whole tumour section area occupied by desmoplastic stroma, was seen in approximately one third of evaluated cases (Table 2).

**Table 2:** Histological parameters under study.

	Mean (SD)	3-year ROC Cut Off (Distribution percentile)
**Whole Section Tumour- Infiltrating Lymphocytes (% of WS tumour area)**		
Mean (SD)	9.29 (5.48)	
*WS Tumour Lymphocytes (% of WS tumour area)*		
	*No of cases (%)*	
1–5%	115 (37.9%)	
6–20%	174 (57.4%)	
20–50%	14 (4.7%)	
*By ROC Cut Off*	*No of cases (%)*	Lymphocytes 5% (30th percentile)
High	188 (62.1%)	
Low	115 (37.9%)	
NR	41	
**Normal Tissue Tumour- Infiltrating Lymphocytes (% of WS tumour area)**		
		
Mean (SD)	2.15 (6.49)	
*WS Normal mucosa Lymphocytes (% of WS tumour area)*	*No of Cases (%)*	
1–5%	199 (73.1%)	
6–50%	73 (26.9%)	
*By ROC Cut Off*	*No of cases (%)*	Lymphocytes 15% (90th percentile)
High	13 (4.8%)	
Low	259 (95.2%)	
NR	72	
**Desmoplastic Stroma (% of Whole Section)**		
Mean (SD)	16.84 (8.55)	
*By ROC Cut Off*	*No of Cases (%)*	20% (70th percentile)
High	117 (46.2%)	
Low	136 (53.8%)	
NR	91	

WS, Whole section; SD, Standard Deviation; ROC, Receiver Operating Characteristic curve analysis, NR. Not recorded

### Gene expression and combined immune response expression clusters

Gene expression data were available in 286 tumours and in 144 matched normal mucosa samples. The expression of genes in tumours was low for CD3Z, CD8, CXCL13, SNAI2 and ESR1 (40-DCq <32) and moderate for IGHM, CD4, CXCL9 and FOXP3 (40-DCq 32–35). Data on gene expression are shown in Table 3.

**Table 3:** mRNA parameters under study.

	Tumour Mean 40-DCq (SD) N = 286	Normal Mucosa Mean 40-DCq (SD) N = 144	Tumour 3-year ROC Cut Off (Distribution percentile)
**CD3Z**	25.56 (5.96)	28.79 (5.70)	27.70 (55th)
**CD8**	25.32 (7.52)	26.15 (7.55)	32.03 (70th)
**CD4**	33.22 (2.87)	33.59 (2.76)	32.69 (30th)
**CXCL9**	34.87 (5.27)	29.76 (7.25)	36.33 (55th)
**CXCL13**	30.30 (5.89)	31.27 (6.74)	28.06 (30th)
**IGHM**	32.13 (7.01)	38.40 (3.25)	33.64 (40th)
**FOXP3**	34.30 (2.24)	32.77 (3.77)	34.71 (55th)
**SNAI2**	30.11 (5.34)	28.69 (5.80)	33.80 (80th)
**ESR1**	24.61 (4.14)	Not done	27.58 (75th)
**Stage_Site_tumour mIS partitioning parameter**	**N = 286**		
Stage II Right Cluster-Low	15 (5.6%)		
Stage II Right Cluster-High	15 (5.6%)		
Stage II Left Cluster-Low	44 (16.5%)		
Stage II Left Cluster-High	20 (7.5%)		
Stage III Right Cluster-Low	33 (12.4%)		
Stage III Right Cluster-High	17 (6.4%)		
Stage III Left Cluster-Low	83 (31.1%)		
Stage III Left Cluster-High	40 (14.9%)		
Missing data	19		

Cq, Cycle quantification; SD, Standard Deviation; ROC, Receiver-Operating Curve; mIS, mRNA-based ImmuneScore; ND, Not done.

**Fig 2:**
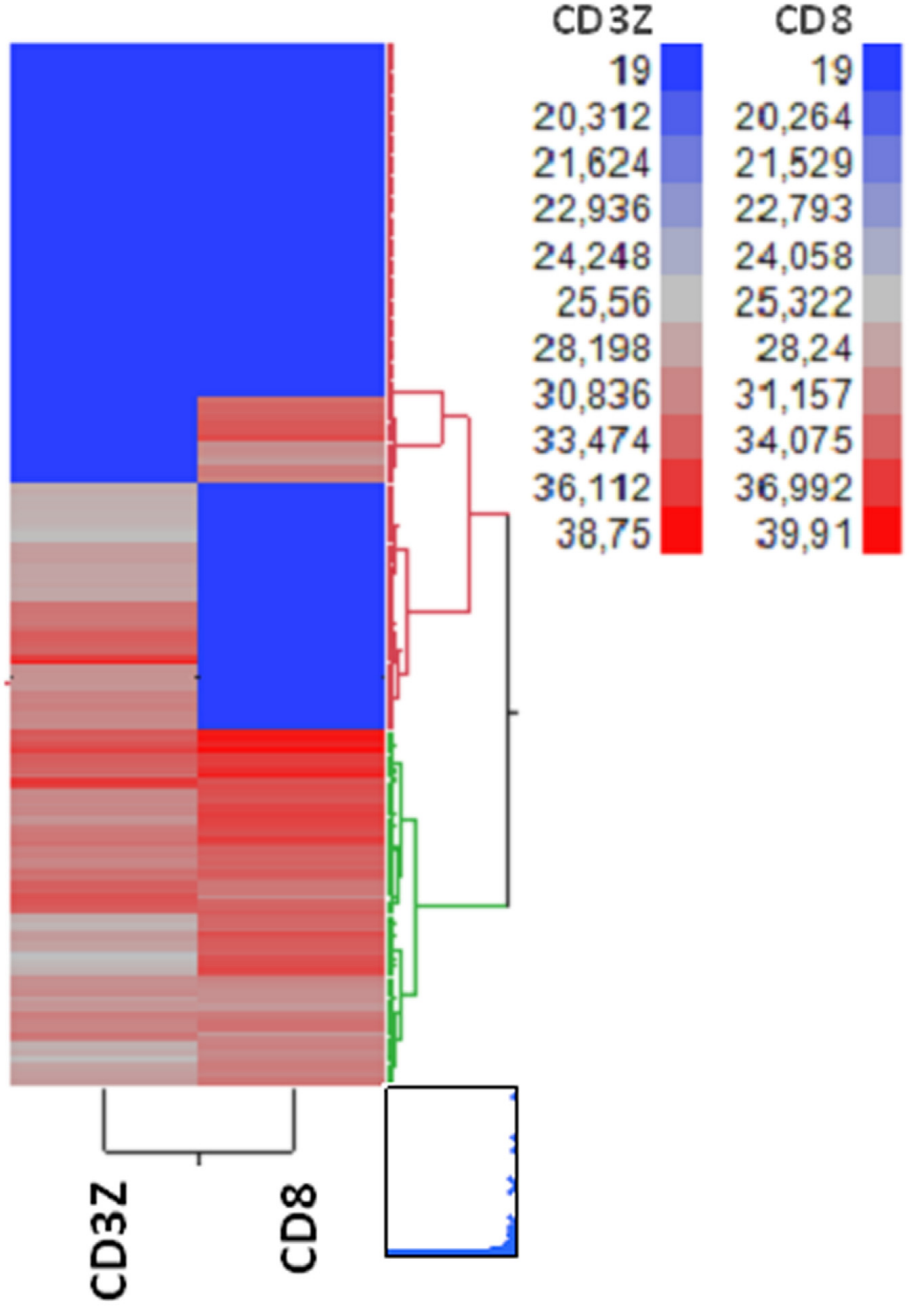
mRNA-based 2-cluster tumour Immune Score heatmap (mIS).

Two-way clustering expression analysis was performed for combinations of the seven immune response genes (CD3Z, CD8, CD4, CXCL9, CXCL13, IGHM and FOXP3) and the resulting clusters were examined for a) variability of gene expression between patient groups, b) optimal separation of patient groups and c) prognostic significance (S2–S4 Figs). Gene expression of CD4 and FOXP3 showed minimal variability between tumours in all examined profilers and were excluded from further cluster analysis. As no multigene profile had prognostic significance in the total number of tumours, criteria a) and b) were used in order to select a 2-cluster ImmunoScore profiler based on the mRNA expression of tumour CD3Z and CD8 (Fig 2). Among 286 informative CRC cases, the mIS showed robust separation of two patient groups (clusters): Cluster 1 (red dendrogram, n = 188) was characterised by relatively low CD3Z and CD8 mRNA expression in the majority of cases and was called mIS-low, while Cluster 2 (green dendrogram, n = 98) contained cases with higher CD3Z and CD8 mRNA expression and was defined as mIS-high. In fact, profilers based on the expression of a higher number of genes (CD3Z, CD8, CXCL9, CXCL13 and IGHM) and consisting of 2, 3 or 4 clusters did not show marked gene expression variability between tumour groups, did not improve separation of clusters nor did they exhibit prognostic significance for patient outcome (S1–S3 Figs). The distribution of mIS clusters by stage and site of CRC is shown in Table 3.

### Univariable prognostic significance of clinicopathological, molecular and immmune response parameters

In univariable analysis, clinical and pathological parameters with prognostic significance for patient outcome were the AJCC-UICC TNM stage (Stage II CRC, Hazard Ratio [HR] for relapse 0.29, 95% Confidence Interval [CI] 0.17–0.49, p = 0.0005, HR for death 0.24, 95% CI 0.12–0.47, Wald’s p = 0.0005) and tumour site for OS only (Left CRC, HR = 0.59, 95% CI 0.38–0.91, p = 0.021

The presence of necrosis at histology was associated with superior outcome; in the absence of tumour necrosis, the HR for relapse was 1.71 (95% CI 1.05–2.71, Wald’s p = 0.033) and the HR for death was 1.98 (95% CI 1.14–3.43, p = 0.012). Patients with dMMR tumours exhibited a numerically, but not significantly, longer DFS (3-year DFS rate 87.9% for dMMR vs 78.4% for pMMR, chi-square test, p = 0.40) and OS (3-year OS rate 90.6% for dMMR vs 87.3% for pMMR, p = 0.33). Neither KRAS exon 2, 3, 4 nor BRAF exon 15 mutations were significantly associated with outcome; although BRAF-mutant CRC was associated with a 75% increased risk of death, the low number of cases precluded it from becoming statistically significant. The percentage of tumour-infiltrating lymphocytes (TIL) per tumour area in representative whole sections was not correlated to DFS or OS, at the examined cut-offs of 5%, 20% and 50% neither at the ROC cut-off at the 30th percentile.

The tumour expression of any of the immune response genes was not found to impact on patient outcome on its own, with the exception of tumour positive CD8 mRNA expression, for which a trend for reduced risk for relapse was seen (HR = 0.51, 95% CI 0.26–0.99, Wald’s p = 0.05). SNAI2 mRNA expression was observed in 56 cases (20%) and was not associated with increased risk for relapse and death. Interestingly, tumour mRNA expression of ESR1 (71 cases, 24.8%) was strongly associated with inferior DFS (HR = 2.33, 95% CI 1.35–4.02, p<0.001) and OS (HR = 1.74, 95% CI 1.02–2.97, p = 0.04).

### Partitioning Analysis in Tumour tissue

We applied partitioning analysis by using the 3-year DFS rate as the response endpoint and patient age (<65 vs ≥65), tumour stage (stage II vs stage III), tumour site (Right vs Left CRC), KRAS mutational status (wild-type vs any exon 2, 3, 4 mutation) and tumour mIS (tumour CD3/CD8 mRNA cluster-low vs cluster-high) as input factors in the 267 patients who had all the above data available. Three classification variables were employed by the recursive partition: stage, site and mIS, separating patients to eight groups with significant differences in DFS (Wald’s p = 0.002) and OS (p<0.0001). The subgroup of patients with stage III Right-sided mIS-high CRC was used as the reference category and was attributed a Hazard Ratio of 1 both for relapse and death (Table 4).

**Table 4:** Prognostic significance of tumour immune response and clinicopathological parameters in univariable analysis.

Parameter	HR for relapse (95% CI)	P-value	HR for death (95% CI)	P-value
***Category*: *Positive***				
**CD3Z**	0.72 (0.42–1.24)	0.24	1.05 (0.64–1.73)	0.85
**CD8**	0.51 (0.26–0.99)	**0.050**	0.90 (0.52–1.56)	0.71
**CD4**	0.70 (0.41–1.21)	0.20	0.66 (0.40–1.11)	0.12
**CXCL9**	0.77 (0.45–1.32)	0.34	0.86 (0.51–1.42)	0.55
**CXCL13**	0.61 (0.35–1.07)	0.08	0.98 (0.55–1.74)	0.95
**IGHM**	0.65 (0.38–1.10)	0.11	0.88 (0.53–1.46)	0.62
**FOXP3**	1.41 (0.83–2.39)	0.21	1.08 (0.65–1.78)	0.77
**SNAI2**	1.40 (0.75–2.62)	0.28	1.39 (0.78–2.49)	0.27
**ESR1**	2.33 (1.35–4.02)	**<0.001**	1.74 (1.02–2.97)	**0.041**
**dMMR**	0.69 (0.28–1.73)	0.4	0.64 (0.26–1.59)	0.33
**BRAF mutation**	1.28 (0.52–3.17)	0.59	1.75 (0.7–4.35	0.23
**KRAS mutation**	1.32 (0.86–2.03)	0.2	1.36 (0.83–2.23)	0.21
**Stage II**	0.29 (0.17–0.49)	**0.0005**	0.24 (0.12–0.47)	**0.0005**
**No necrosis at histology Whole section TIL**	1.71 (1.05–2.71)	**0.033**	1.98 (1.14–3.43)	**0.012**
**1–5%**	1			
**6–20%**	1.42	0.24	1.42	0.21
**>20%**	3.18	0.12	2.48	0.22
**ROC cut off: High vs Low**	1.57 (0.98–2.53)	0.063	1.45 (0.84–2.53)	0.19
***Stage_Site_tumour mISpartitioning parameter***		**0.002**		**<0.0001**
**Stage II Right Cluster-Low**	0.26 (0.03–2.51)	0.24	0.12 (0.01–0.94)	0.041
**Stage II Right Cluster-High**	0.23 (0.02–2.25)	0.21	0.10 (0.001–10)	0.98
**Stage II Left Cluster-Low**	0.26 (0.05–1.26)	0.092	0.12 (0.03–0.48)	0.001
**Stage II Left Cluster-High**	0.76 (0.17–3.40)	0.72	0.36 (0.10–1.22)	0.10
**Stage III Right Cluster-Low**	**2.28 (1.05–8.02)**	**0.023**	1.26 (0.52–3.06)	0.61
**Stage III Right Cluster-High**	1		1	
**Stage III Left Cluster-Low**	1.13 (0.34–3.79)	0.84	0.48 (0.2–1.12)	0.094
**Stage III Left Cluster-High**	0.95 (0.26–3.53)	0.94	0.50 (0.19–1.32)	0.16

**Fig 3:**
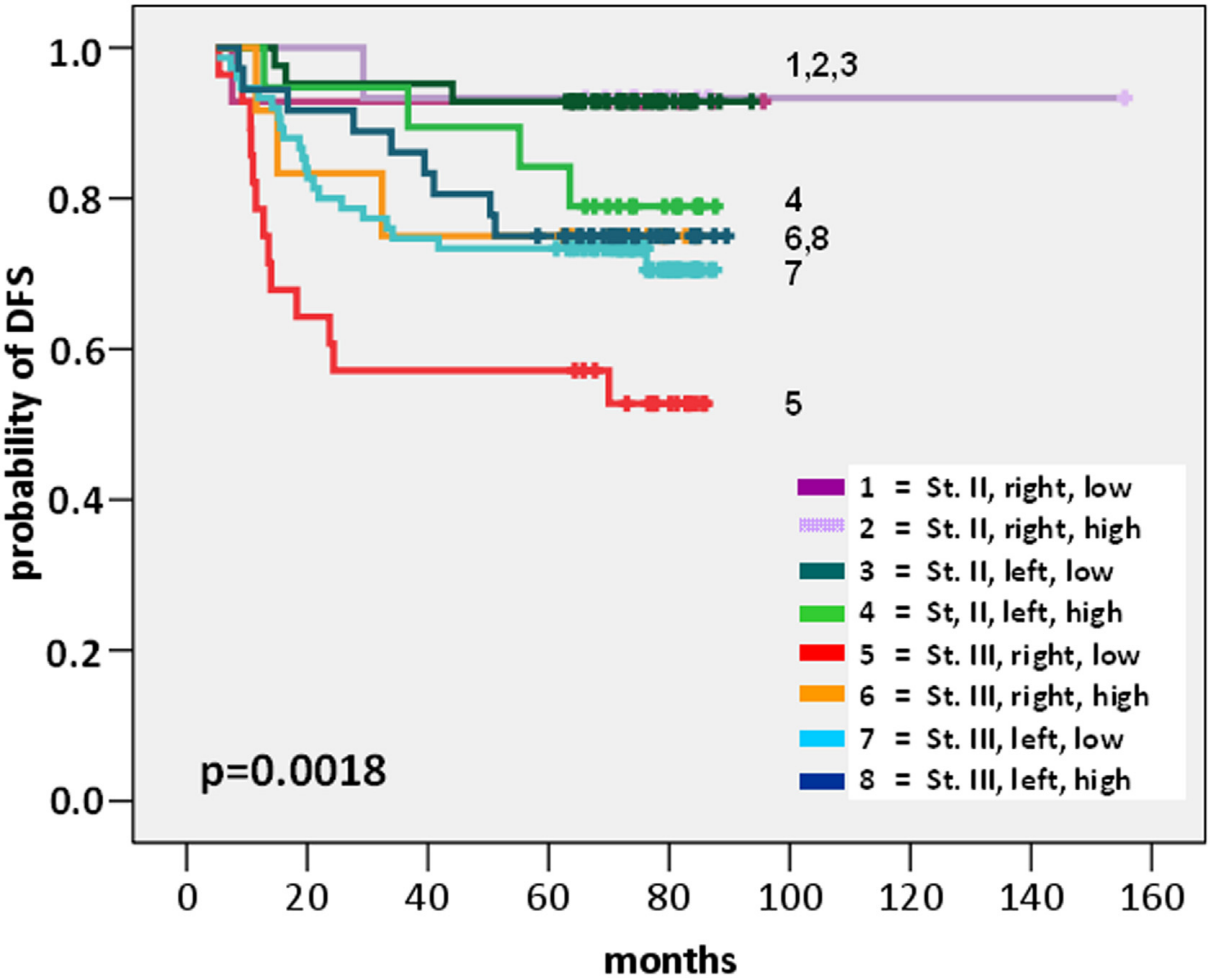
Disease-free survival of Stage_Site_ tumour mIS groups.

**Fig 4:**
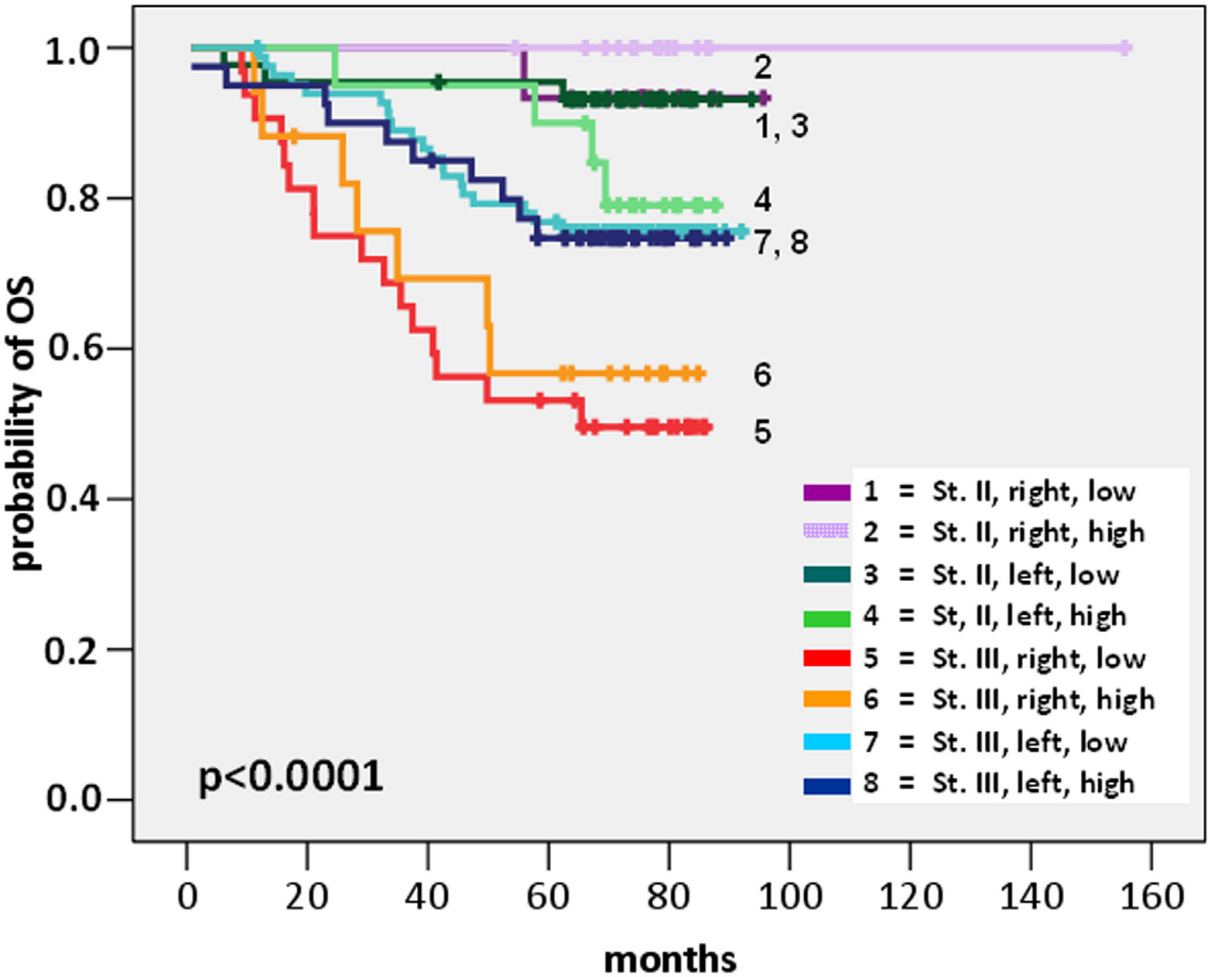
Overall survival of Stage_Site_tumour mIS groups.

For patients with stage II CRC, tumour mIS was not prognostic, neither in left nor in right colon. However, among patients with stage III CRC, tumour mIS carried prognostic significance in right-sided, though not in left-sided tumours. Specifically, for patients with right-sided stage III CRC, mIS had a favourable prognostic significance: 3-year DFS rate was 75% in mIS-high versus 57.1% in mIS-low cases (HR for relapse 2.28 in mIS-low compared to the reference category of mIS-high cases, 95% CI 1.05–8.02, p = 0.023). In contrast, in patients with left-sided stage III CRC, no outcome difference was seen between tumour mIS clusters: the 3-year DFS rate was 86.1% in mIS-high versus 77.4% in mIS-low cases. The site-specific prognostic impact of tumour mIS for stage III CRC was lost in the OS analyses. However, few deaths had occurred in the already small patient subgroups examined, resulting in low power for detection of moderate survival differences. The relative risks for relapse and death of the partitioning patient subgroups in comparison to the Stage III Right-sided mIS-high reference category are summarised in Table 4, while the survival curves are visualised in Figs 3 and 4.

In multiple imputation with 100 imputed data sets at 5% significance level, ESR1 repeated the prognostic findings of DFS only, but not OS, while the complex partitioning parameter Stage_Site_tumour mIS repeated the findings of DFS, OS (results shown in S1 Statistical Analyses).

**Fig 5:**
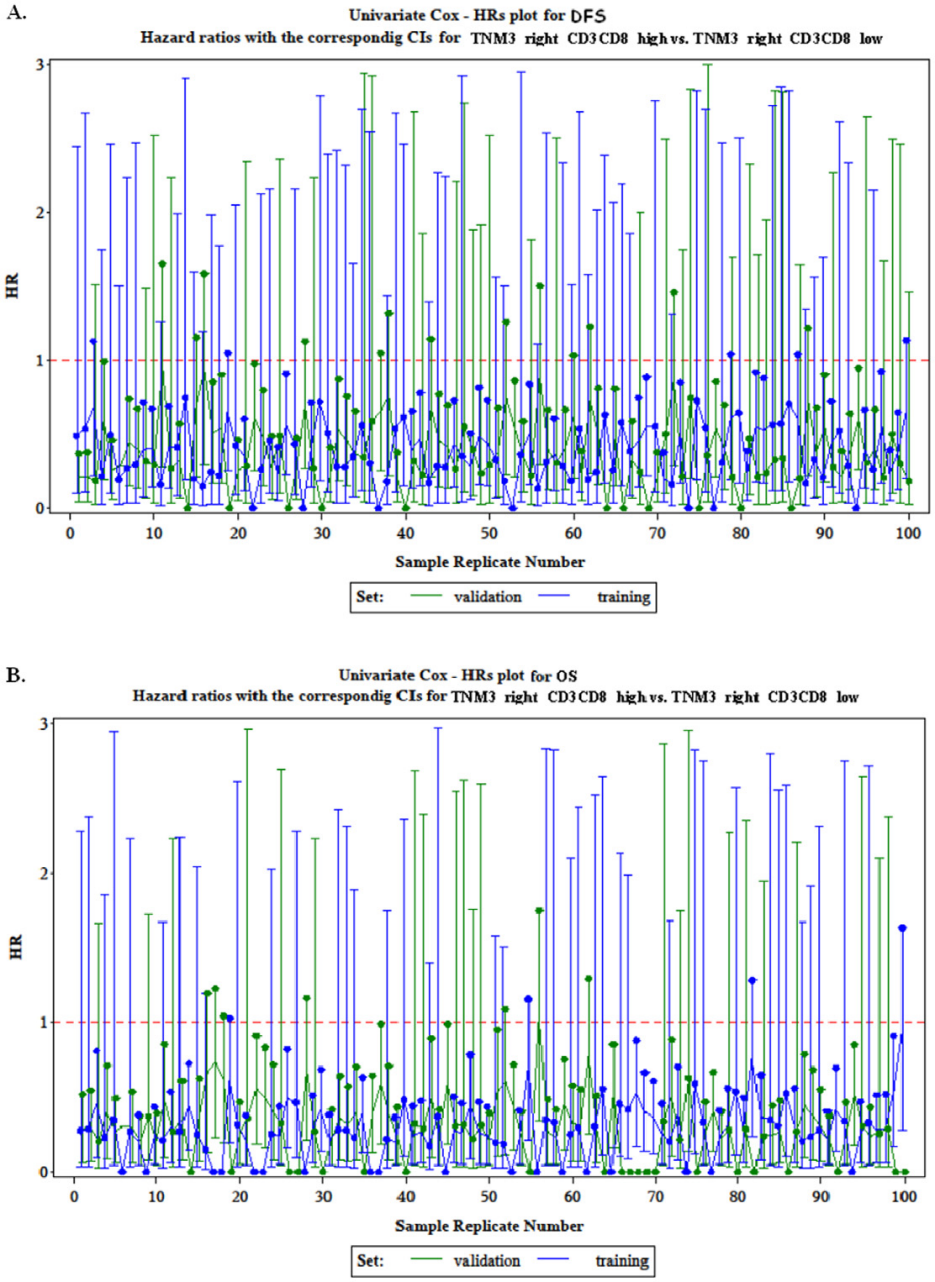
Hazard Ratios for relapse (a) and death (b).

In order to examine the robustness of our partitioning analysis and the validity of the prognostic significance of tumour mIS in patients with stage III right-sided colon cancer, we split the relevant patient cohort to training and validation sets randomly for 100 iterations. We observed that in the vast majority of cases, the hazard ratios for relapse and death were <1 for mIS-high cases compared to mIS-low in stage III right-sided CRC (Fig 5). The hazard ratios were compared in tumour mIS-high versus tumour mIS-low cases in stage III right-sided colon cancer patients, in randomly generated training and validation sets). \In contrast, in the same cross-validation test, in patients with stage III left-sided CRC there was no consistent pattern for the hazard ratios for relapse and death to be <1 in mIS-high cases.

### Correlations and Interactions

The presence of moderate/strong tumour lymphocytic infiltration was significantly associated with age ≥65 (70.5% in ≥65 vs 56.4% in <65, p = 0.013), presence of symptoms at diagnosis (82.3% in PS 1 vs 60.3% in PS 0, p = 0.034) and possibly with right-sided tumours: moderate or strong lymphocytic infiltration was seen in 64 (68.8%) of R CRC compared to 122 (58.7%) of L CRC tumours (p = 0.09). Of interest, no associations were seen between tumour lymphocytic infiltration and KRAS or BRAF mutational status, neither with mRNA expression of any of the immune response genes (CD3Z, CD8, CD4, CXCL9, CXCL13, IGHM and FOXP3), SNAI2 or ESR1. Correlations, which were statistically significant and of potential biologicalal relevance were observed in immune response gene expression: the tumour mRNA expression of CD3Z was associated with CD8 (Spearmann Rho 0.45, p = 0.0001).

### Expression and prognostic significance of parameters under study in Normal Colonic Mucosa

Several molecular and pathological characteristics were studied in normal mucosa adjacent to the tumours. In matched comparisons of tumour vs normal mucosa cases (n = 272), lymphocytic infiltration was less intense in normal mucosa (Mean Difference of Infiltrating Lymphocytes as % of the section area: +7.6% in favour of tumour, p<0.0001). More than 5% lymphocytes in normal mucosa were observed only in 73 cases (26.9%) and had no prognostic significance for DFS or OS (HR = 0.25, 95% CI 0.04–1.83, Wald’s p = 0.17 and HR = 0.20, 95% CI 0.03–1.45, p = 0.98, respectively). Lymphocytes infiltrating normal mucosa were not significantly associated with mRNA gene expression in normal mucosa of any of the immune response genes, SNAI2 or ESR1. In paired comparisons of tumour vs normal mucosa IR gene expression (n = 144), there was a trend for higher expression of CXCL9 (Wilcoxon test, p = 0.05) and lower expression of CD3Z, CXCL13 and IGHM in the malignant tissue.

The 2-cluster profiler based on the mRNA expression in normal mucosa of CD3Z and CD8 (Normal mIS, S4 Fig) consisted of one homogeneous cluster with relatively high expression of CD3Z and CD8 (n = 59) and a second heterogeneous cluster with low CD8 but variable CD3Z expression (n = 70). The Normal mIS failed to show any prognostic significance for DFS (p = 0.74) or OS (p = 0.89).

We applied the same partitioning analysis by using the 3-year DFS rate as the response endpoint for patient age, tumour stage, tumour site (Right vs Left CRC), KRAS mutational status and Normal Mucosa mIS (CD3/CD8 mRNA cluster-variable vs cluster-high) as input factors in 126 patients for whom all data were available from normal mucosa samples. Three classification variables were employed by the CART partition: stage, site and Normal Mucosa mIS. The partitioning classification separated patients to eight groups with overall significant differences in DFS (p = 0.001) and in OS (p<0.0001). However, the statistical significance was mainly driven by the tumour stage and site. The immune response in normal mucosa had no prognostic significance, even in patients with stage III right-sided colon cancer: patients with right-sided stage III tumours who belonged to the Normal Mucosa mIS-high group had similar survival compared to those of the Normal mucosa mIS-variable group (3-year DFS was 64.5% for Normal mucosa mIS-high vs 61.0% for Normal Mucosa mIS-variable). The risk for relapse in various stage- and site-specific subgroups by Tumour mIS versus Normal Mucosa mIS are summarised in the S3 Table.

### Multivariable Analysis

**Fig 6:**
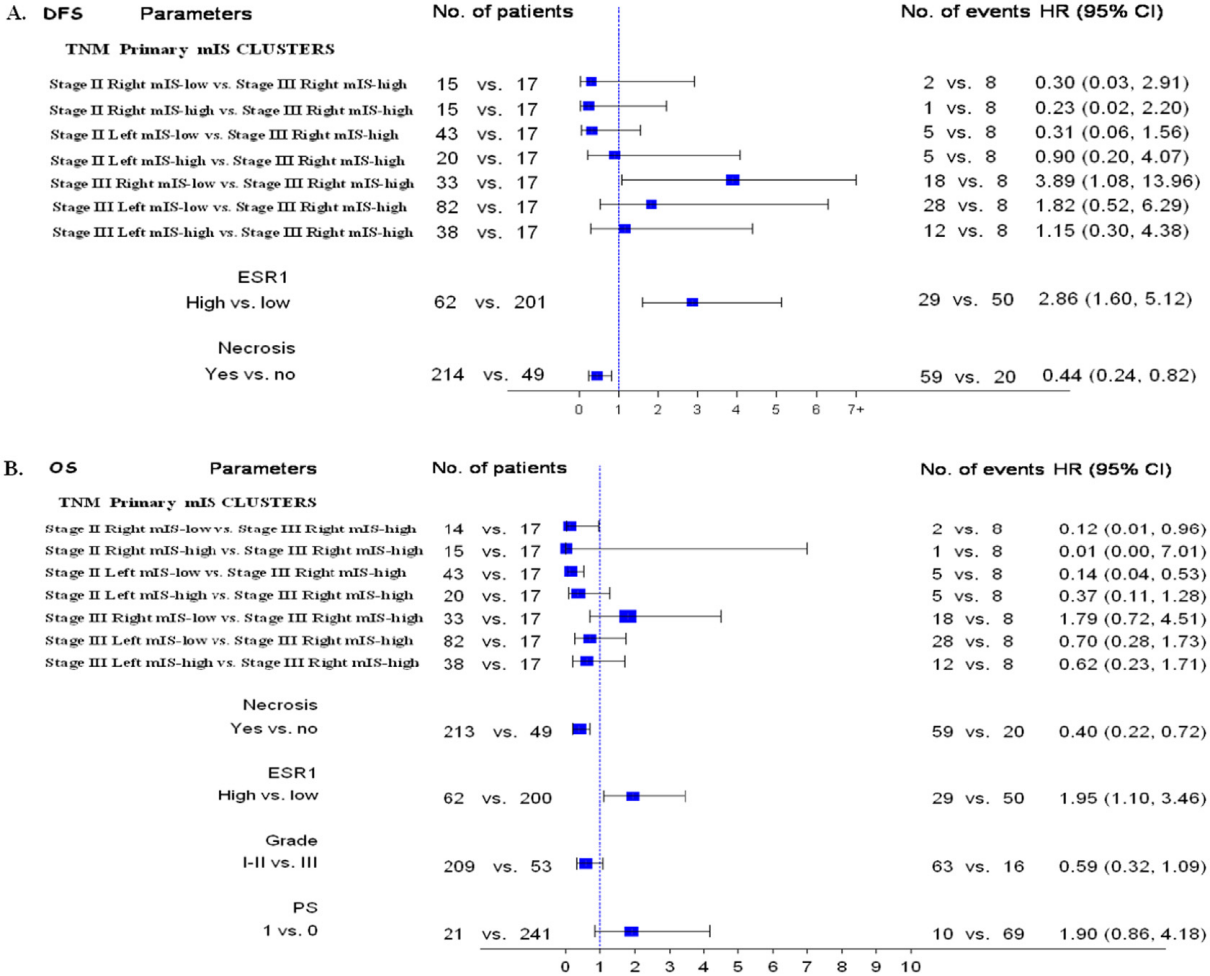
Multivariate analysis Forrest Plots for risk for relapse (a) and death (b).

In multivariable analysis, 226 patients had all relevant data available for the calculation of the independent risk for relapse and 251 patients for the risk for death. The presence of tumour necrosis at histology was significantly associated with a reduced risk for relapse (HR = 0.44, 95% CI 0.24–0.82, Wald’s p = 0.01) and death (HR = 0.40, 95% CI 0.22–0.72, p = 0.002). Tumour ESR1 mRNA expression was an adverse prognostic factor, independently correlating with relapse (HR = 2.86, 95% CI 1.60–5.12, p = 0.0004) and death (HR = 1.95, 95% CI 1.10–3.46, p = 0.022). The complex partitioning parameter Stage_Site_tumour mIS was also significantly and independently associated with DFS (Wald’s p = 0.0005) and OS (p = 0.0006). Specifically, in patients with stage III right-sided colon cancer, a low tumour mIS was significantly associated with inferior DFS (HR = 3.89, 95% CI 1.08–13.96, p = 0.037) and non-significantly with inferior OS (HR = 1.79, 95% CI 0.72–4.51, p = 0.21, Fig 6)

## Discussion

The deficiencies of the currently used TNM staging system have been emphasized by several investigators; its prognostic inaccuracy may be due to its focus on tumour characteristics only, while ignoring host mechanisms at play. The host immune reaction that infiltrates the tumour (tumour-associated immune contexture) is emerging as an important factor for defining not only prognosis, but also possibly response to chemotherapy and therapeutic antibodies[12, 13]. Pioneering work by Galon et al has established the Immunoscore, a semi-quantitative immunohistochemical characterisation of the type (CD3+ and CD8 or CD45RO+), number and localization (centre of tumour and invasive margin) of host immune cells as a reliable prognostic factor[14]. In fact, Bindea et al reported that the Immunoscore retained independent prognostic significance in multivariate analysis of 599 colorectal cancer patients, while the TNM staging system did not[15]. A recent meta-analysis of 120 trials studying the prognostic impact of host immune response in various solid tumours concluded that there is a universal beneficial impact of immune infiltrates with cytotoxic and memory T cell phenotype, while the role of B cells, NK cells, Tregs, Th2 cells and macrophages depends on cancer type and stage[16]. The choice of immune response genes to be studied in our cohort was based on published research establishing their regulatory or effector roles.[3, 16, 17]

In our series of patients with resected stage II/III CRC, we have used qRT-PCR relative quantification of mRNA expression of seven immune response genes from TMA cores taken from the centre of tumour and the invasive margin. In keeping with accumulating evidence from IHC Immunoscore studies, we have identified CD3Z and CD8 as the most biologically relevant genes that separated two tumour clusters: one with low and one with high mRNA expression, possibly reflecting tumour infiltration by T lymphocytes with a cytotoxic, memory and Th1 phenotype. As previously published, immune infiltrates of the B, NK, Th2, Treg or macrophage lineage have lesser prognostic impact. Moreover,functionally diverse subsets of these cell populations have different biologic relevance and prognostic significance[3, 18]. Our inability to confirm the latter may be due to our inability to reliably identify these cell subsets. Finally, the expression of some immune genes, if tightly correlated to that of CD3 and CD8, may be redundant to monitor.

The choice of the qRT-PCR methodology and that of summing mRNA RQ values from both CT and IM tissue cores (mRNA-based Immunoscore, mIS) seems counter-intutive but may have some advantages. IHC is subjected to bias, subjectivity, interobserver disagreement (due to pre-analytical and analytical problems), inherent complexity and protocol variability[19]. As put by Galon et al, standardisation and harmonisation of IHC protocol procedures is necessary before clinical application[14]. Some of these issues stand for qRT-PCR as well, however RQ provides a dynamic, objective range of measurements not prone to observer bias. Provided tumour macrodissection is standardized for reliable TCC, a validated qRT-PCR assay for could result in more reliable and objective data on tumour immune infiltration. Our use of clustered CD3 and CD8 RQ values from CT and IM cores does indeed preclude us from obtaining information on the role of the immune response in each of these two tumour tissue compartments, however it is not different from the IHC Immunoscore strategy of calculating a total score from the addition of the IHC immune infiltrates in CT and IM[12]. Bindea et al reported that T-cell responses are prognostic in both the CT and IM of CRC[15]. We fully acknowledge the fact that validation and generation of useful information require: a) comparison of the IHC Immunoscore and mIS in a larger CRC cohort for data concordance and prognostic significance, b) comparison of the mIS in whole section versus TMA core samples, c) evaluation of the impact on mIS mRNA data of factors, such as tissue necrosis, tumour cell content and cellularity, tissue type, hemorrhage and macrodissection biases, d) validation in an independent cohort. We plan to implement these in our HE6C08 cohort of 800 patients with stage II/III CRC.

In view of the functional diversity of the immune cell repertoire, indistinguishable on H&E morphology, it was expected that lymphocytic infiltrates had no prognostic significance in our series. Opposing biological functions, including tumour-promoting versus tumour-suppressive effects, have been reported by several investigators for Treg cells, CD4+ T cells,macrophages and NK cells, according to their distinct functional subsets[2, 20]. The novelty of our findings can be summarised in the prognostic impact of a CD3/CD8 mRNA immune response that differs according to colon cancer stage and colonic site. We found tumour-associated immune response (mIS) to be protective from disease relapse only in patients with right-sided stage III colon cancer, as opposed to stage II disease or left-sided stage III CRC, in which it had no prognostic significance. No overall survival differences were seen though, this could be due to the small size of the patient subgroups examined and the presence of highly effective therapies for relapsed colon cancer.

The right colon differs from the left in several aspects, such as embryological origin, vasculature, histological characteristics, mutational and gene expression profiles and environmental factors (toxins, flora and diet). R colon tumours bear more commonly BRAF mutations and the MMR phenotype, though the low incidence in our series makes them an unlikely culprit[8, 21, 22]. As many as 202 genes with more than two-fold difference in expression between R and L colon were identified, among whom some point to a more immune response-permissive environment in the right colon[23]. Right-sided colonic tumours bore increased expression of genes regulating anti-tumour immune reactivity (HOX genes, CD44, CD68, CD163, CD3Z, TNFR, LYN, LCK, NOS, TRAF and FLT4) and reduced expression of genes orchestrating tumour hijack of immune responses in order to promote inflammation, tumour proliferation and immune evasion (PD1, CDX2, ERK, APC, b-catenin, Epithelial Mesenchymal Transition mediators, BCL2 and IL4, 5, 7 and 17). Bendardaf et al found high IHC VEGF-A expression, a potent immunosuppressor, to be common in Left (61%), but less so in Right colon cancer (45%)[24]. Fu et al examined 1324 CRC tumours and found the incidence of lymphoid follicles to be higher in R colon cancer, while Kaz et al reported differences in the methylation status of 8388 genetic loci in R versus L colonic mucosa, some related to immune function[25, 26]. Therefore, it is possible that differences in the microenvironment along with the host immune system profiles orchestrate an immune response that is effectively tumour-suppressive when active in the right colon, but not in the left. Similarly, the stage-specificity of the immune response actually leads us to a question of essential biology: are stages II and III of colon cancer serial steps in the progression of the malignancy or distinct diseases with different biologies? Indeed, Bindea et al depicted an «Immune Landscape» of CRC by means of microarray and IHC and reported that in 107 tumours studied, each Immune Response profile represented a transition pattern associated with tumour stage[27]. This could mean either that an immune response has different characteristics and effects in different tumour stages, or alternatively that the stage of a tumour is inherently linked to a specific biology and host immune reaction. Di Caro et al found a different prognostic effect of tertiary lymphoid structures and CD3+ lymphocytes in stage II compared to stage III colon cancer patients[28].

Another important finding of our study is the lack of prognostic significance of the immune response in the adjacent to the tumour normal mucosa. Indeed, Sherwood et al used IHC and T-cell receptor sequencing to establish different immune responses in CRC compared to adjacent normal mucosa, the tumour lymphocytic infiltrates being characterised by less diversity and more clonality and activation[17]. A practical implication of our finding lies in the need for appropriate processing and selection of tumour sections along with careful macrodissection before assaying the tumour-associated immune contexture.

Histological necrosis and ESR1 mRNA expression were independent and universal prognosticators in our series. The presence of tumour necrosis was associated with superior outcome. Contradictory reports of both favourable and adverse prognostic significance have been generated for various solid tumours. Different causes of necrosis may have a distinct prognostic impact: rapid tumour proliferation, hypoxia and migratory potential would argue for adverse, while inadequate vascularisation, functional apoptosis and effective host immune response for favourable [29]. Therefore, proper identification of functionally different «necrotic» entities is needed. ESR1 was shown to function as a pro-proliferative, pro-angiogenic, pro-migratory and anti-apoptotic oncogene in muscle-invasive bladder, gallbladder, lung and prostate cancer[30–33]. Tsiambas et al reported ESR1 gene copy number gains in 8.3% of 60 CRC patients[34]. Other groups studying CpG methylation profiles reported that upon CRC progression, the ESR1 gene effecting proliferation is demethylated, while the pro-apoptotic ESR2 gene is gradually methylated[35]. Although preclinical data suggest an immune-regulatory role for ESR1, its low expression and the moderate sample size precluded us from studying correlations of its expression with immune gene expression in our CRC cohort.

To conclude, we present here translational research data from which we generate novel hypotheses warranting further validation: a) we propose a mRNA-based CD3/CD8 immunoscore, potentially suitable for the development of a certified In Vitro Test, b) we propose a differential prognostic impact of the immune response in different stages and sites of CRC (protective in Stage III right-sided colon cancer only), c) we suggest the need to profile the immune response in appropriately macrodissected tumour tissue only and not adjacent normal mucosa and d) we propose a novel marker, ESR1 mRNA expression, with potential usefulness as a prognosticator, as well as a therapeutic target in CRC.

## Supporting Information

S1 CONSORT Checklist CONSORT checklist. (DOC)

S1 Fig Three-cluster profiler of all Immune Response genes in tumour. (TIF)

S2 Fig Three-cluster profiler of Immune Response genes in tumours with the exclusion of CD4 and FOXP3. (TIF)

S3 Fig Two-cluster profiler based on tumour CD3Z, CD8, CXCL13. (TIF)

S4 Fig Two-cluster profiler based on mRNA expression of CD3Z and CD8 in Normal Mucosa. (TIF)

S1 Statistical Analyses A, Multiple Imputation Report. B, Comparison of observed survival to predicted survival by quantiles of the predicted risk. C, Multivariate models without the complex Partitioning parameter.
(DOC)

S1 Table Primers and probes for KRAS and BRAF mutation testing. (DOCX)

S2 Table Primer and probe sequences used for quantitative reverse transcription-polymerase chain reaction (qRT-PCR) assessment. (DOCX)

S3 Table Hazard Ratios for Relapse by stage and site using the Tumour mIS versus the Normal Mucosa mIS. (DOCX)

S4 Table Fuctions of the studied genes in the immune response. (DOCX)
