# Supplementary figures and images for: Immune Response Gene Expression in Colorectal Cancer Carries Distinct Prognostic Implications According to Tissue, Stage and Site: A Prospective Retrospective Translational Study in the Context of a Hellenic Cooperative Oncology Group Randomised Trial
Source: PLoS One. 2015 May 13;10(5):e0124612. doi: 10.1371/journal.pone.0124612 (PMC4430485; doi:10.1371/journal.pone.0124612)

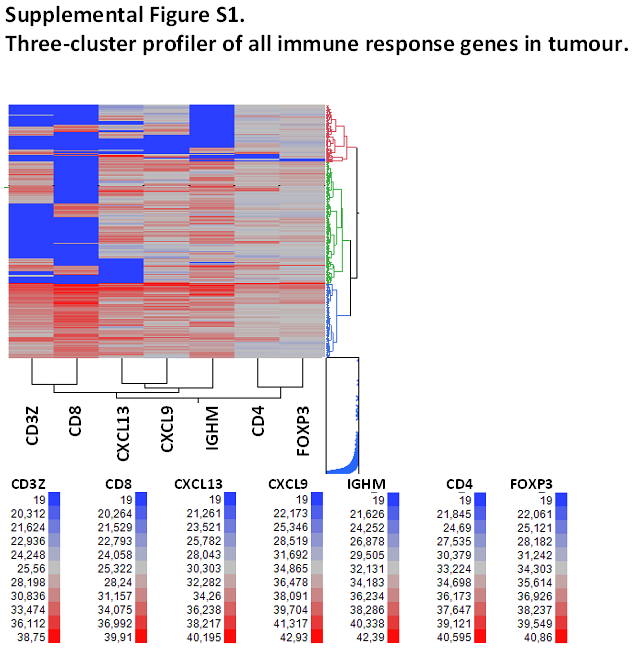

Supplement: S1 Fig — (TIF) [file pone.0124612.s002.tif]

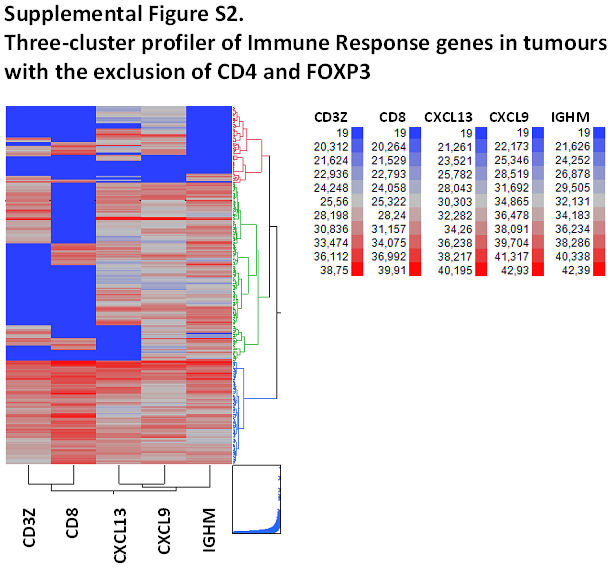

Supplement: S2 Fig — (TIF) [file pone.0124612.s003.tif]

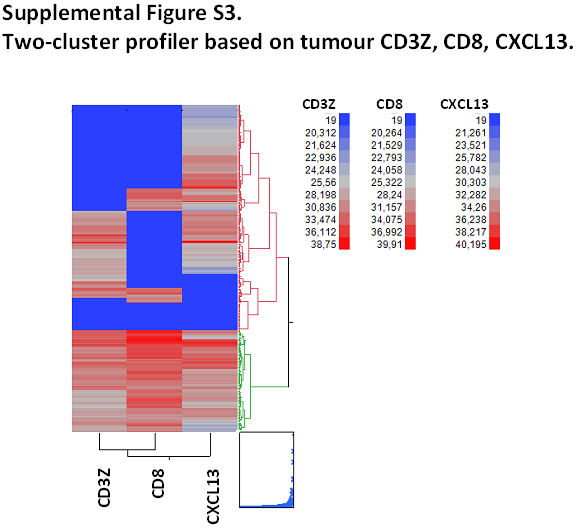

Supplement: S3 Fig — (TIF) [file pone.0124612.s004.tif]

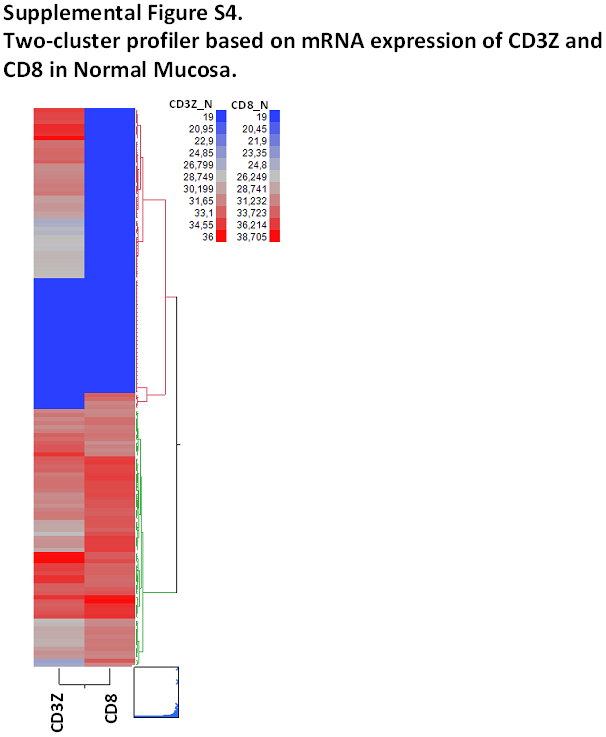

Supplement: S4 Fig — (TIF) [file pone.0124612.s005.tif]
