# Supplementary material for: Immune Response Gene Expression in Colorectal Cancer Carries Distinct Prognostic Implications According to Tissue, Stage and Site: A Prospective Retrospective Translational Study in the Context of a Hellenic Cooperative Oncology Group Randomised Trial
Source: PLoS One. 2015 May 13;10(5):e0124612. doi: 10.1371/journal.pone.0124612 (PMC4430485; doi:10.1371/journal.pone.0124612)
Supplement: S1 Statistical Analyses — A, Multiple Imputation Report. B, Comparison of observed survival to predicted survival by quantiles of the predicted risk. C, Multivariate models without the complex Partitioning parameter. (DOC) [file pone.0124612.s006.doc]

# A. Multiple Imputation Report

*We have performed a multiple imputation analysis for the mRNA parameters. Basic patient demographics and tumour characteristics were used as auxiliary variables.*

*The MCMC algorithm was implemented producing 100 imputed data sets.*

*Table 1* *depicts the difference for each mRNA parameter between the mean of the initial dataset and the mean of the 100 imputed data sets.* Figure 1 *presents the distribution of the mRNA parameters mean value among the 100 imputed data sets.*

*The results of the imputation exercise were applied on the ESR1 and complex partitioning parameter findings of the manuscript. In particular, we have reported the overexpression of ESR1 as a significant unfavorable prognostic factor in terms of OS and DFS. Additionally, we identified the complex partitioning parameter as a significantly associated factor with OS and DFS.*

***Our findings were:***

*ESR1*

*The univariate Cox regression analysis (with the cutoff at the 75^th^ percentile) for OS was statistically significant for the 9 out of 100 datasets, while for DFS for the 96 out of 100 at the 5% level of significance.*

*At the 1% level of significance, 1 out of 100 datasets was significant for OS and 34 out of 100 for DFS.*

*Complex partitioning parameter*

*The clustering procedure was not repeated for each of the 100 imputed datasets, but the clustering variable (mIS) was created based on the way the initial clustering has partitioned the levels of expression of CD3Z and CD8.*

*For both OS and DFS, in univariate Cox, the parameter retained significance in 100 out of 100 datasets (Table 2) at the 5% level of significance. When the 1% level of significance was assessed only 5 out of 100 datasets for DFS reached significance, while OS was not affected.*

| Table 1: Imputed data differences in mean from the initial dataset | | | |
| --- | --- | --- | --- |
|  | **Mean Value** | |  |
| **mRNA parameter** | **initial dataset of 344** | **after multiple imputation (n = 100)** | **Difference**  **(initial -imputed)** |
| CD3Z | 25.557 | 25.821 | 0.263 |
| CD4 | 33.115 | 33.126 | 0.011 |
| CD8 | 25.088 | 25.438 | 0.350 |
| CXCL13 | 30.257 | 30.320 | 0.064 |
| CXCL9 | 34.849 | 34.839 | -0.010 |
| ESR1 | 24.602 | 24.734 | 0.132 |
| FOXP3 | 34.221 | 34.226 | 0.005 |
| IGHM | 32.068 | 32.108 | 0.040 |
| SNail2 | 30.064 | 30.128 | 0.064 |

| **Table 2:** Percent of imputed datasets that the univariate COX model reached significance | | | |
| --- | --- | --- | --- |
|  | **Level of significance** | **OS** | **DFS** |
| ESR1 – 75^th^ perc. | 5% | 9/100 | 96/100 |
| ESR1 – 75^th^ perc. | 1% | 1/100 | 34/100 |
| Complex partitioning parameter | 5% | 100/100 | 100/100 |
| Complex partitioning parameter | 1% | 100/100 | 5/100 |

| Figure 1: Distribution of the imputed mRNA data |
| --- |
|  |

**B. Comparison of observed survival to predicted survival by quantiles of the predicted risk.**

The following graphs compare observed vs. mean predicted survival for DFS and OS at specific time points, where adequate number of events was obtained. 95% CL for the mean predicted values are presented with the error bars.

**Observed vs. Predicted**

**C. Multivariate models without the complex Partitioning parameter**

The following table presents the multivariable analysis results without the clustering analysis. In the case of OS, histological grade did not retain its significance.

The high low classification based on the cd8 and cd3 levels is a useful predictor, when assessed as a complex parameter with tumour stage and site. The way this high-low classification contributes to prognosis does not alter the effect of tumour stage and site or vice versa (2-sided p>0.05 for Cox with interaction testing, both for OS and DFS). On the contrary, it intensifies the prognostic ability of stage and site by resulting in more separated survival curves. The analysis conducted by multiple imputation validated this finding, with all of the imputed datasets used resulting in retaining the complex parameter’s prognostic significance.

Multivariable analysis table

| **Overall survival** | **Hazard Ratio** | **95% CI** | **Wald’s p-value** |
| --- | --- | --- | --- |
| ESR1 |  |  |  |
| High vs. Low | 1.92 | 1.10-3.35 | 0.0222 |
|  |  |  |  |
| Necrosis |  |  |  |
| Yes vs. No | 0.40 | 0.23-0.72 | 0.0023 |
|  |  |  |  |
| PS |  |  |  |
| 1 vs. 0 | 2.10 | 0.97-4.55 | 0.0614 |
|  |  |  |  |
| AJCC-UICC stage |  |  |  |
| III vs. II | 6.56 | 2.90-14.86 | <0.0001 |
|  |  |  |  |
| Primary Site |  |  |  |
| left vs. right | 0.61 | 0.35-1.04 | 0.0705 |
| **Disease-free survival** | **Hazard Ratio** | **95% CI** | **Wald’s p-value** |
| ESR1 |  |  |  |
| High vs. Low | 2.72 | 1.55-4.79 | 0.0005 |
|  |  |  |  |
| Necrosis |  |  |  |
| Yes vs. No | 0.46 | 0.25-0.85 | 0.0132 |
|  |  |  |  |
| AJCC-UICC stage |  |  |  |
| III vs. II | 4.82 | 2.23-10.40 | <0.0001 |
