# Supplementary material for: Immune Response Gene Expression in Colorectal Cancer Carries Distinct Prognostic Implications According to Tissue, Stage and Site: A Prospective Retrospective Translational Study in the Context of a Hellenic Cooperative Oncology Group Randomised Trial
Source: PLoS One. 2015 May 13;10(5):e0124612. doi: 10.1371/journal.pone.0124612 (PMC4430485; doi:10.1371/journal.pone.0124612)
Supplement: S1 Table — (DOCX) [file pone.0124612.s007.docx]

**Supplemental Table S1. Primers and probes for KRAS and BRAF mutation testing.**

| **Gene, coding seq** | **Primer** | **Sequence (5'-3')** | **Sequenced region coordinates (GRCh38)** | | **Length** |
| --- | --- | --- | --- | --- | --- |
| KRAS, exon 2 | F | CGTCTGCAGTCAACTGGAATTT |  |  |  |
|  | R | AAAGAATGGTCCTGCACCAGTAA |  |  |  |
|  | FN* | TTAACCTTATGTGTGACATGTTCTAA | chr12:25245428 | chr12:25245453 | 174 |
|  | RN* | TGGTCCTGCACCAGTAATATGC | chr12:25245233 | chr12:25245254 |  |
| KRAS, exon 3 | F | AGGTGCACTGTAATAATCCA |  |  |  |
|  | R | ATGGCATTAGCAAAGACTC |  |  |  |
|  | FN | TGCACTGTAATAATCCAGA | chr12:25227433 | chr12:25227451 | 200 |
|  | RN | ATTATATTCAATTTAAACCCAC | chr12:25227212 | chr12:25227233 |  |
| KRAS, exon 4 | F | GGTTTTGAAAGATATTTGT |  |  |  |
|  | R | TTTTATTTCCTAGTATAGCAT |  |  |  |
|  | FN | TTACTAATGACTGTGCTAT | chr12:25225794 | chr12:25225812 | 229 |
|  | RN | GATATATTAAATGACATAACAG | chr12:25225544 | chr12:25225565 |  |
| BRAF, exon 15 | F | ATAATGCTTGCTCTGATAGG |  |  |  |
|  | R | GTGAATACTGGGAACTATGAA |  |  |  |
|  | FN | CTACTGTTTTCCTTTACTTAC | chr7:140753404 | chr7:140753424 | 256 |
|  | RN | GGGAACTATGAAAATACTATA | chr7:140753168 | chr7:140753188 |  |
| none | M13F | GTAAAACGACGGCCAGT |  |  |  |
|  | M13R | CAGGAAACAGCTATGACC |  |  |  |
