# Supplementary material for: Immune Response Gene Expression in Colorectal Cancer Carries Distinct Prognostic Implications According to Tissue, Stage and Site: A Prospective Retrospective Translational Study in the Context of a Hellenic Cooperative Oncology Group Randomised Trial
Source: PLoS One. 2015 May 13;10(5):e0124612. doi: 10.1371/journal.pone.0124612 (PMC4430485; doi:10.1371/journal.pone.0124612)
Supplement: S2 Table — (DOCX) [file pone.0124612.s008.docx]

**Supplemental Table S2. Primer and probe sequences used for quantitative reverse transcription-polymerase chain reaction (qRT-PCR) assessment.**

| **PrimerName** | **GenSymbol** | **NM_Nr** | **Probe Name** | **Probe Sequence** | **Forward_Name** | **Forward_Sequence** | **Rev_Name** | **Rev_Sequence** |
| --- | --- | --- | --- | --- | --- | --- | --- | --- |
| **MP456** | ESR1 | NM_000125 | **MP456** | ATGCCCTTTTGCCGATGCA | **MP456_For** | GCCAAATTGTGTTTGATGGATTAA | **MP456_Rev** | GACAAAACCGAGTCACATCAGTAATAG |
| **MP428** | SNAI2 | NM_003068 | **MP428** | ATACAGTGATTATTTCCCCGTATCTCT | **MP428_For** | CAAACTACAGCGAACTGGACACA | **MP428_Rev** | TGACAGGCATGGAGTAACTCTCA |
| **MP317** | CD3Z_CD247 | NM_198053 | **MP317** | CAAAGCTCTGTGCCTCTGTAATCGGCA | **MP317_For** | CACCGCGGCCATCCT | **MP317_Rev** | AGTTTGGGATCCAGCAGGC |
| **MP606** | CD4 | NM_001195017 | **MP606** | ACATCAAGGTTCTGCCCACATGGTCCACCC | **MP606-For** | GTGGCAGTGTCTGCTGAGTGA | **MP606-Rev** | AGCACAATCAGGGCCATTG |
| **MP607** | CD8 | NM_001145873 | **MP607** | TTCCTGCCAGCGAAGCCCAC | **MP607-For** | TGAGCAACTCCATCATGTACTTCAG | **MP607-Rev** | GGCGCCGGTGTTGGT |
| **MP494** | CXCL9 | NM_002416 | **MP494** | AAAGGGTCGCTGTTCCTGCATCAGC | **MP494_For** | TGCAAGGAACCCCAGTAGTGA | **MP494_Rev** | GGTGGATAGTCCCTTGGTTGG |
| **MP498** | CXCL13 | NM_006419 | **MP498** | TGGTCAGCAGCCTCTCTCCAGTCCA | **MP498_For** | CGACATCTCTGCTTCTCATGCT | **MP498_Rev** | AGCTTGTGTAATAGACCTCCAGAACA |
| **MP551** | FOXP3 | NM_014009 | **MP551** | TTTTCTGTCAGTCCACTTCACCAAGCCTG | **MP551_FOR** | CCCACAAGCCAGGCTGAT | **MP551_REV** | GCATCGGGTCCTTGTCCA |
