# Supplementary material for: Immune Response Gene Expression in Colorectal Cancer Carries Distinct Prognostic Implications According to Tissue, Stage and Site: A Prospective Retrospective Translational Study in the Context of a Hellenic Cooperative Oncology Group Randomised Trial
Source: PLoS One. 2015 May 13;10(5):e0124612. doi: 10.1371/journal.pone.0124612 (PMC4430485; doi:10.1371/journal.pone.0124612)
Supplement: S3 Table — (DOCX) [file pone.0124612.s009.docx]

Supplemental Table S3. Hazard Ratios for Relapse by stage and site using the Tumour mIS versus the Normal Mucosa mIS.

| **Stage Site mIS partitioning parameter** |  |  |  |  |  |
| --- | --- | --- | --- | --- | --- |
| ***Tumour mIS*** | **HR for relapse** | p-value= 0.0018 | ***Normal Mucosa mIS*** | **HR for relapse** | p-value= 0.0013 |
| **Stage II Right Cluster-Low** | 0.26 | 0.24 |  | 0.13 | 0.05 |
| **Stage II Right Cluster-High** | 0.23 | 0.21 |  | 0.09 | 0.02 |
|  |  |  |  |  |  |
| **Stage II Left Cluster-Low** | 0.26 | 0.09 |  | 0.24 | 0.01 |
| **Stage II Left Cluster-High** | 0.76 | 0.72 |  | 0.34 | 0.08 |
|  |  |  |  |  |  |
| **Stage III Right Cluster-Low** | 2.28 | 0.02 |  | 1.21 | 0.67 |
| **Stage III Right Cluster-High** | 1 |  |  | 1 |  |
|  |  |  |  |  |  |
| **Stage III Left Cluster-Low** | 1.13 | 0.84 |  | 0.63 | 0.22 |
| **Stage III Left Cluster-High** | 0.95 | 0.94 |  | 0.59 | 0.18 |
|  |  |  |  |  |  |
