# Supplementary material for: Immune Response Gene Expression in Colorectal Cancer Carries Distinct Prognostic Implications According to Tissue, Stage and Site: A Prospective Retrospective Translational Study in the Context of a Hellenic Cooperative Oncology Group Randomised Trial
Source: PLoS One. 2015 May 13;10(5):e0124612. doi: 10.1371/journal.pone.0124612 (PMC4430485; doi:10.1371/journal.pone.0124612)
Supplement: S4 Table — (DOCX) [file pone.0124612.s010.docx]

**Supplemental Table S4. Fuctions of the studied genes in the immune response.**

| ***Immune Response Gene (Locus)*** | ***Function*** |
| --- | --- |
|  |  |
| CD3Z 1q24.2 | The protein encoded by this gene is T-cell receptor zeta which forms the T-cell receptor-CD3 complex. The zeta chain plays an important role in coupling antigen recognition to several intracellular signal-transduction pathways. Low expression of the antigen results in impaired immune response. Marker of activated T lymphocytes. |
|  |  |
| **CD8**  2p12 | The CD8 antigen is a cell surface glycoprotein found on most cytotoxic T lymphocytes that mediates efficient cell-cell interactions with class I MHC antigen-presenting cells. |
|  |  |
| **CD4**  12p13.31 | This gene encodes a membrane glycoprotein of T-helper lymphocytes that interacts with MHC class II antigens in APCs. This gene is expressed not only in T-helper lymphocytes, but also in B cells, macrophages and functions to initiate or augment the early phase of T-cell activation |
|  |  |
| **CXCL9**  4q21 | This T-helper type 1 chemokine is involved in T cell trafficking and activates anti-tumour T-cellular responses. |
|  |  |
| **CXCL13**  4q21 | B lymphocyte chemoattractant. It is a CXC chemokine strongly expressed in the follicles of the spleen, lymph nodes, and Peyer's patches that preferentially promotes the migration of B lymphocytes and activates humoral immune response. |
|  |  |
| **IGHM**  14q32.33 | The IGHM gene encodes the C region of the mu heavy chain, which defines the IgM isotype. Mature B cells express IgM and IgD immunoglobulins which are complexed with Ig-alpha and Ig-beta to form the B cell receptor (BCR). Binding of an antigen to the immunoglobulin activates a humoural immune response. |
|  |  |
| **FOXP3** Xp11.23 | The protein encoded by this gene is a member of the forkhead/winged-helix family of transcriptional regulators and is considered to be a marker of immunosuppressive T-regulatory lymphocytes that suppress and keep cellular immune responses in check. |
